# Pumice as a Novel Natural Heterogeneous Catalyst for the Designation of 3,4-Dihydropyrimidine-2-(1*H*)-ones/thiones under Solvent-Free Conditions

**DOI:** 10.3390/molecules27186044

**Published:** 2022-09-16

**Authors:** Hany M. Abd El-Lateef, Mohamed Gouda, Mai M. Khalaf, Saad Shaaban, Nadia A. A. Elkanzi, El Sayed A. Saber, Antar A. Abdelhamid, Ali M. Ali

**Affiliations:** 1 Department of Chemistry, College of Science, King Faisal University, Al-Ahsa 31982, Saudi Arabia; 2 Department of Chemistry, Faculty of Science, Sohag University, Sohag 82534, Egypt; 3 Chemistry Department, Faculty of Science, Mansoura University, Mansoura 35516, Egypt; 4 Chemistry Department, College of Science, Jouf University, Sakaka P.O. Box 2014, Saudi Arabia; 5 Chemistry Department, Faculty of Science, Aswan University, Aswan P.O. Box 81528, Egypt; 6 Geology Department, Faculty of Science, Sohag University, Sohag 82534, Egypt; 7 Chemistry Department, Faculty of Science, Albaha University, Albaha P.O. Box 1988, Saudi Arabia

**Keywords:** pumice catalyst, one-pot reaction, 3,4-dihydropyrimidine-2(1*H*)-ones/thiones, pore size, heterogeneous catalyst

## Abstract

In this study, pumice is used as a novel natural heterogeneous catalyst for the synthesis of 3,4-dihydropyrimidine-2-(1*H*)-ones/thiones via the one-pot multi-component condensation of aromatic aldehydes, urea/thiourea, and ethyl acetoacetate or acetylacetone in excellent yields (up to 98%). The physical and chemical properties of the catalyst were studied. Their geochemical analysis revealed a basaltic composition. Furthermore, X-ray diffraction showed that it is composed of amorphous materials with clinoptilolite and heulandites zeolite minerals in its pores. Moreover, pumice has a porosity range from 78.2–83.9% (by volume) and is characterized by a mesoporous structure (pore size range from 21.1 to 64.5 nm). Additionally, it has a pore volume between 0.00531 and 0.00781 m^2^/g and a surface area between 0.053 and 1.47 m^2^/g. The latter facilitated the reaction to proceed in a short time frame as well as in excellent yields. It is worth noting that our strategy tolerates the use of readily available, cheap, non-toxic, and thermally stable pumice catalyst. The reactions proceeded smoothly under solvent-free conditions, and products were isolated without tedious workup procedures in good yields and high purity. Indeed, pumice can be reused for at least five reuse cycles without affecting its activity.

## 1. Introduction

The multi-component approach is a crucial synthetic strategy in organic chemistry via giving access to different heterocyclic compounds like imidazoles, pyrazoles, pyridines, and acridines [1,2,3,4,5]. The Biginelli reaction is considered the most common multi-component reaction and is used to synthesize dihydropyrimidinones. The latter exhibit an extensive range of pharmaceutical and biological effectiveness, such as antitumor, antiviral, anti-inflammatory, and antibacterial properties [6]. Furthermore, dihydropyrimidinones are also considered potential calcium channel blockers [7], neuropeptide antagonists, α1a–adrenergic antagonists, and antihypertensive agents. Moreover, 2-oxodihydropyrimidine-5-carboxylate was isolated from numerous natural marine products [8], such as the batzelladine alkaloids, which are considered potent HIV gp-120-CD_4_ inhibitors [9,10]. In general, the Biginelli reaction requires a long reaction time (≥24 h) and affords low yields, particularly in the case of substituted aldehydes [11,12]. Therefore, the Biginelli reaction is continuing to attract the attention of scientists to develop more efficient procedures for synthesizing dihydropyrimidinones. Within this context, many catalysts, and a plethora of reagents and methods, were explored to design and prepare dihydropyrimidinones [13,14]; however, much concern has been directed to the conduction of the Biginelli reaction under solvent-free conditions. The latter included amberlyst-15, Nafion-H, KSF clay with dry acetic acid under microwave irradiation [10], ionic liquids (e.g., *n*-butyl-3-methylimidazolium tetrafluoroborate and hexafluorophosphorate) [15,16], and ultrasonication using ceric ammonium nitrate [17]. Lewis acids (e.g., Fe(CF_3_CO_2_)_3_), cerium(III) trislaurylsulfonate), in combination with transition metals and a suitable proton source [18], lanthanide triflates (e.g., Yb(OTf)_3_) [19], lanthanide chloride [20], and indium chloride (e.g., YbCl_3_) [21] were also explored.

Despite the considerable success of these methods, they are limited with respect to the reagent cost, tedious workup procedures, and long reaction time. In this context, the Biginelli reaction still requires an efficient protocol for synthesizing pyrimidinone compounds.

Noteworthily, porous materials manifested great importance in the catalysis field. These included microporous compounds (e.g., zeolites, MOFs, and zeotypes) and mesoporous and microporous materials (e.g., mesostructured silicas, mesoporous zeolites, and aluminas) [22,23]. Furthermore, the channels and cavities of the porous materials can selectively separate ions and molecules according to their different sizes, which can be used in many applications, such as energy efficiency and catalysis [24]. Pumice is used as a raw material in several fields owing to its porous structure, which improves the selectivity and yields of the reactions [25,26,27,28].

Recently, the Biginelli reaction was employed to construct dihydropyrimidinones/thiones [29] under solvent-free and catalyst-free conditions using various types of β-ketoesters [30].

In this work, pumice was used as a green, novel, and natural catalyst in synthesizing 3,4-dihydropyrimidine-2(1*H*)-ones/thiones via the one-pot multi-component condensation of aromatic aldehydes, urea/thiourea, and β-ketoesters. The chemical composition, crystal structure, and physical properties (surface area, pore volume, porosity, pore size) of pumice as porous catalysis have been measured, as well as the catalytic effect of pumice.

## 2. Experimental

All reagents were purchased from Fluka (Buchs, Switzerland), Aldrich (St. Louis, MO, USA), and Merck (Kenilworth, NJ, USA). All reactions were checked by thin-layer chromatography (TLC) using silica gel plates G/UV-254 of 0.25-mm thickness (Merck 60F254) and UV light (254 nm/365 nm) for visualization. Melting points were measured with a Kofler melting point apparatus (Weinheim, Germany) and uncorrected. IR spectra were recorded with an FTIR Alpha Bruker Platinum ATR (Billerica, MA, USA). ^1^H-NMR and ^13^C-NMR spectra were recorded in DMSO-d_6_ or CDCl_3_ at 400 and 100 MHz, respectively, on a Bruker Bio Spin AG spectrometer. Elemental analyses were obtained on a Perkin-Elmer CHN-analyzer model (Waltham, MA, USA).

### 2.1. General Procedure for Synthesis of 3,4-Dihydropyrimidine-2(1H)-ones/thiones **2a,b**–**19a,b**

A mixture of aromatic aldehyde (e.g., benzaldehyde (10 mmol, 1.06 g), *p*-chlorobenzaldehyde (10 mmol, 1.46 g), *p*-nitrobenzaldehyde (10 mmol, 1.51 g), *p*-methoxybenzaldehyde (10 mmol, 1.36 g), *N,N*-dimethylaminobenzaldehyde (10 mmol, 1.49 g) or 4-hydroxybenzaldehyde (10 mmol, 1.22), ethyl acetoacetate (15 mmol, 1.95 mL) or acetyl acetone (15 mmol, 1.54 mL), and urea (11 mmol, 0.66 g)/thiourea (11 mmol, 0.84 g) was heated at 180°C while stirring (under solvent-free conditions) in the presence of pumice catalyst (0.4 g) for 1–3 min. After completion of the reaction, the hot reaction mixture was poured into 10 mL ethanol. The catalyst was recovered in this case as solid precipitate, which was directly filtered. The filtrate was kept at room temperature for a few hours, and the formed precipitate was filtered to yield the desired products.

#### 2.1.1. Ethyl 6-methyl-2-oxo-4-phenyl-1,2,3,4-tetrahydropyrimidine-5-carboxylate (**2a**)

M.P. 202 °C; IR: 3181, 3157 (2 NH), 1735 (C=O ester), 1650 (C=O cyclic) cm^−1^; ^1^H NMR (DMSO-*d_6_*): δ 9.13, 7.68 (s, 2H, 2NH exchanged by D_2_O), 7.33–7.25 (m, 5H, CH_arom_), 5.17 (s, 1H, CH_Cyclic_), 4.01–3.99 (q, *J* = 7.1 Hz, 2H, CH_2_CH_3_), 2.26 (s, 3H, CH_3Pyrimidinium_), 1.11–1.10 (t, *J* = 7.0 Hz, 3H, CH_2_CH_3_); ^13^C NMR (DMSO-*d_6_*): δ 165.82, 152.59, 148.75, 145.34, 128.82, 127.69, 126.70, 99.83, 59.63, 54.47, 18.22, 14.52. (*Anal*. Calcd. For C_14_H_16_N_2_O_3_ (260.28): C, 64.60; H, 6.20; N, 10.76. Found: C, 64.56; H, 6.07; N, 10.54 (Figures S1–S3; Supplementary Materials).

#### 2.1.2. Ethyl 4-(4-chlorophenyl)-6-methyl-2-oxo-1,2,3,4-tetrahydropyrimidine-5-carboxylate (**3a**)

M.P. 211 °C; IR: 3166, 3113 (2 NH), 1740 (C=O ester), 1641 (C=O cyclic) cm^−1^; ^1^H NMR (DMSO-*d_6_*): δ 9.40, 7.99 (s, 2H, 2NH exchanged by D_2_O), 7.47–7.29 (m, 4H, CH_arom_), 5.13 (s, 1H, CH_Cyclic_), 4.01–3.98 (q, *J* = 7.1 Hz, 2H, CH_2_CH_3_), 2.21 (s, 3H, CH_3Pyrimidinium_), 1.09–1.08 (t, *J* = 7.0 Hz, 3H, CH_2_CH_3_); ^13^C NMR (DMSO-*d_6_*): δ 167.14, 151.42, 146.41, 141.91, 130.04, 126.25, 125.71, 98.15, 55.14, 53.54, 19.37, 13.29. (*Anal*. Calcd. For C_14_H_15_ClN_2_O_3_ (294.73): C, 57.05; H, 5.13; N, 9.50. Found: C, 56.89; H, 5.02; N, 9.24.

#### 2.1.3. Ethyl 6-methyl-4-(4-nitrophenyl)-2-oxo-1,2,3,4-tetrahydropyrimidine-5-carboxylate (**4a**)

M.P. 209 °C; IR: 3191, 3159 (2 NH), 1749 (C=O ester), 1671 (C=O cyclic) cm^−1^; ^1^H NMR (DMSO-*d_6_*): δ 10.20, 8.19 (s, 2H, 2NH exchanged by D_2_O), 7.90–7.04 (m, 4H, CH_arom_), 5.29 (s, 1H, CH_Cyclic_), 4.09–3.99 (q, *J* = 7.1 Hz, 2H, CH_2_CH_3_), 2.07 (s, 3H, CH_3Pyrimidinium_), 1.10–1.08 (t, *J* = 7.0 Hz, 3H, CH_2_CH_3_); ^13^C NMR (DMSO-*d_6_*): δ 168.45, 151.54, 149.87, 145.13, 128.76, 127.16, 126.38, 100.04, 58.41, 54.13, 19.13, 14.14. (*Anal*. Calcd. For C_14_H_15_N_3_O_5_ (305.28): C, 55.08; H, 4.95; N, 13.76. Found: C, 54.96; H, 4.71; N, 13.61.

#### 2.1.4. Ethyl 4-(4-methoxyphenyl)-6-methyl-2-oxo-1,2,3,4-tetrahydropyrimidine-5-carboxylate (**5a**)

M.P. 201–202 0C; IR: 3113, 3101 (2 NH), 1713 (C=O ester), 1647 (C=O cyclic) cm^−1^; ^1^H NMR (DMSO-d6): δ 9.09, 7.61 (s, 2H, 2NH exchanged by D2O), 7.17–6.87 (m, 5H, CH_arom_), 5.11 (s, 1H, CH_Cyclic_), 4.00–3.99 (q, *J* = 7.1 Hz, 2H, CH_2_CH_3_), 3.73 (qs, 3H, OCH_3_), 2.75 (s, 3H, CH_3Pyrimidinium_), 1.13–1.10 (t, *J* = 7.0 Hz, 3H, CH_2_CH_3_); ^13^C NMR (DMSO-d_6_): δ 165.85, 158.94, 152.61, 148.41, 137.55, 127.85, 114.19, 100.12, 59.58, 55.54, 53.84, 18.20. (*Anal.* Calcd. For C_15_H_18_N_2_O_4_ (290.31): C, 62.06; H, 6.25; N, 9.65. Found: C, 61.81; H, 6.13; N, 9.39 (Figures S4–S6; Supplementary Materials).

#### 2.1.5. Ethyl 4-[4-(dimethylamino)phenyl]-6-methyl-2-oxo-1,2,3,4-tetrahydropyrimidine-5-carboxylate (**6a**)

M.P. 233 °C; IR: 3178, 3151 (2 NH), 1713 (C=O ester), 1641 (C=O cyclic) cm^−1^; ^1^H NMR (DMSO-*d_6_*): δ 9.74, 7.99 (s, 2H, 2NH exchanged by D_2_O), 7.72–7.15 (m, 4H, CH_arom_), 5.02 (s, 1H, CH_Cyclic_), 4.01–3.99 (q, *J* = 7.1 Hz, 2H, CH_2_CH_3_), 4.21 (s, 6H, N(CH_3_)_2_), 2.14 (s, 3H, CH_3Pyrimidinium_), 1.10–1.09 (t, *J* = 7.0 Hz, 3H, CH_2_CH_3_); ^13^C NMR (DMSO-*d_6_*): δ 165.85, 152.54, 148.79, 145.37, 128.82, 127.64, 126.79, 99.81, 59.66, 54.48, 40.53, 18.11, 14.24. (*Anal*. Calcd. For C_16_H_121_N_3_O_3_ (260.28): C, 63.35; H, 6.98; N, 13.85. Found: C, 63.25; H, 6.77; N, 13.74.

#### 2.1.6. Ethyl 4-(4-hydroxyphenyl)-6-methyl-2-oxo-1,2,3,4-tetrahydropyrimidine-5-carboxylate (**7a**)

M.P. 232 °C; IR: 3181, 3157 (2 NH), 1735 (C=O ester), 1650 (C=O cyclic) cm^−1^; ^1^H NMR (DMSO-*d_6_*): δ 9.13, 7.68 (s, 2H, 2NH exchanged by D_2_O), 7.33–7.25 (m, 5H, CH_arom_), 5.17 (s, 1H, CH_Cyclic_), 4.01–3.99 (q, *J* = 7.1 Hz, 2H, CH_2_CH_3_), 2.26 (s, 3H, CH_3Pyrimidinium_), 1.11–1.10 (t, *J* = 7.0 Hz, 3H, CH_2_CH_3_); ^13^C NMR (DMSO-*d_6_*): δ 167.29, 150.48, 147.94, 146.14, 128.41, 127.54, 125.78, 99.47, 58.71, 53.15, 17.25, 14.24. (*Anal*. Calcd. For C_14_H_16_N_2_O_4_ (276.28): C, 60.86; H, 5.84; N, 10.14. Found: C, 60.51; H, 6.07; N, 10.03.

#### 2.1.7. Ethyl 6-methyl-4-phenyl-2-thioxo-1,2,3,4-tetrahydropyrimidine-5-carboxylate (**2b**)

M.P. 207–209 °C; IR: 3157, 3143 (2 NH), 1731 (C=O ester) cm^−1^; ^1^H NMR (DMSO-*d_6_*): δ 10.27, 9.59 (s, 2H, 2NH exchanged by D_2_O), 7.35–7.24 (m, 5H, CH_arom_), 5.2 (s, 1H, CH_Cyclic_), 4.02 (q, *J* = 7.1 Hz, 2H, CH_2_CH_3_), 2.30 (s, 3H, CH_3Pyrimidinium_), 1.11 (t, *J* = 7.1 Hz, 3H, CH_2_CH_3_); ^13^C NMR (DMSO-*d_6_*): δ 174.80, 165.62, 145.42, 143.96, 129.00, 128.12, 126.84, 101.29, 60.04, 54.55, 17.61, 14.46. (*Anal*. Calcd. for C_14_H_16_N_2_O_2_S (276.35): C, 60.85; H, 5.84; N, 10.14. Found: C, 60.71; H, 5.81; N, 10.01 (Figures S7–S9; Supplementary Materials).

#### 2.1.8. Ethyl 4-(4-chlorophenyl)-6-methyl-2-thioxo-1,2,3,4-tetrahydropyrimidine-5-carboxylate (**3b**)

M.P. 207–209 °C; IR: 3187, 3155 (2 NH), 1714 (C=O ester) cm^−1^; ^1^H NMR (DMSO-*d_6_*): δ 10.84, 9.41 (s, 2H, 2NH exchanged by D_2_O), 7.81–7.15 (m, 4H, CH_arom_), 5.13 (s, 1H, CH_Cyclic_), 4.26 (q, *J* = 7.1 Hz, 2H, CH_2_CH_3_), 2.24 (s, 3H, CH_3Pyrimidinium_), 1.10 (t, *J* = 7.0 Hz, 3H, CH_2_CH_3_); ^13^C NMR (DMSO-*d_6_*): δ 175.15, 167.64, 147.48, 145.43, 129.41, 127.86, 124.74, 101.14, 60.17, 54.47, 17.65, 14.56. (*Anal*. Calcd. for C_14_H_15_ClN_2_O_2_S (310.80): C, 54.10; H, 4.86; N, 9.01. Found: C, 54.01; H, 4.51; N, 9.11.

#### 2.1.9. Ethyl 6-methyl-4-(4-nitrophenyl)-2-thioxo-1,2,3,4-tetrahydropyrimidine-5-carboxylate (**4b**)

M.P. 207 °C; IR: 3145, 3149 (2 NH), 1738 (C=O ester) cm^−1^; ^1^H NMR (DMSO-*d_6_*): δ 10.15, 9.42 (s, 2H, 2NH exchanged by D_2_O), 7.81–7.02 (m, 4H, CH_arom_), 5.17 (s, 1H, CH_Cyclic_), 4.18 (q, *J* = 7.1 Hz, 2H, CH_2_CH_3_), 2.37 (s, 3H, CH_3Pyrimidinium_), 1.00 (t, *J* = 7.0 Hz, 3H, CH_2_CH_3_); ^13^C NMR (DMSO-*d_6_*): δ 178.84, 169.41, 145.41, 143.93, 129.09, 128.10, 126.14, 107.84, 68.17, 54.74, 17.51, 15.75. (*Anal*. Calcd. for C_14_H_15_N_3_O_4_S (321.35): C, 52.33; H, 4.70; N, 13.08. Found: C, 52.21; H, 4.61; N, 13.01.

#### 2.1.10. Ethyl 4-(4-methoxyphenyl)-6-methyl-2-thioxo-1,2,3,4-tetrahydropyrimidine-5-carboxylate (**5b**)

M.P. 153 °C; IR: 3141, 3112 (2 NH), 1719 (C=O ester) cm^−1^; ^1^H NMR (DMSO-*d_6_*): δ 10.20, 9.30 (s, 2H, 2NH exchanged by D_2_O), 7.47–7.05 (m, 4H, CH_arom_), 5.6 (s, 1H, CH_Cyclic_), 4.02 (q, *J* = 7.1 Hz, 2H, CH_2_CH_3_), 3.95 (s, 3H, OCH_3_), 2.27 (s, 3H, CH_3Pyrimidinium_), 1.10 (t, *J* = 7.0 Hz, 3H, CH_2_CH_3_); ^13^C NMR (DMSO-*d_6_*): δ 175.09, 167.63, 146.45, 144.97, 129.18, 128.73, 126.94, 101.14, 62.16, 55.49, 54.15, 17.08, 15.11. (*Anal*. Calcd. for C_15_H_18_N_2_O_3_S (306.38): C, 58.80; H, 5.92; N, 9.14. Found: C, 58.72; H, 5.81; N, 9.25.

#### 2.1.11. Ethyl 4-[4-(dimethylamino)phenyl]-6-methyl-2-thioxo-1,2,3,4-tetrahydropyrimidine-5-carboxylate (**6b**)

M.P. 209 °C; IR: 3150, 3147 (2 NH), 1747 (C=O ester) cm^−1^; ^1^H NMR (DMSO-d_6_): δ 10.75, 9.57 (s, 2H, 2NH exchanged by D_2_O), 7.34–7.20 (m, 4H, CH_arom_), 5.24 (s, 1H, CH_Cyclic_), 4.44 (s, 6H, N(CH_3_)_2_), 4.03 (q, *J* = 7.1 Hz, 2H, CH_2_CH_3_), 2.12 (s, 3H, CH_3Pyrimidinium_), 1.08 (t, *J* = 7.1 Hz, 3H, CH_2_CH_3_); ^13^C NMR (DMSO-d_6_): δ 177.55, 167.91, 146.14, 144.15, 129.46, 127.11, 125.87, 101.37, 64.15, 55.59, 17.38, 14.75. (*Anal.* Calcd. for C_16_H_21_N_3_O_2_S (319.42): C, 60.16; H, 6.63; N, 13.16. Found: C, 60.22; H, 6.52; N, 13.04.

#### 2.1.12. Ethyl 4-(4-hydroxyphenyl)-6-methyl-2-thioxo-1,2,3,4-tetrahydropyrimidine-5-carboxylate (**7b**)

M.P. 203 °C; IR: 3174, 3140 (2 NH), 1736 (C=O ester) cm^−1^; ^1^H NMR (DMSO-*d_6_*): δ 10.01, 9.74 (s, 2H, 2NH exchanged by D_2_O), 8.46 (s, 1H, OH exchanged by D_2_O), 7.74–7.27 (m, 4H, CH_arom_), 5.2 (s, 1H, CH_Cyclic_), 4.18 (q, *J* = 7.1 Hz, 2H, CH_2_CH_3_), 2.27 (s, 3H, CH_3Pyrimidinium_), 1.17 (t, *J* = 7.0 Hz, 3H, CH_2_CH_3_); ^13^C NMR (DMSO-*d_6_*): δ 175.91, 166.21, 143.15, 141.03, 128.47, 127.10, 126.04, 101.02, 60.75, 54.79, 17.28, 14.91. (*Anal*. Calcd. for C_14_H_16_N_2_O_2_S (292.35): C, 57.52; H, 5.52; N, 9.58. Found: C, 57.83; H, 5.44; N, 9.51.

#### 2.1.13. Methyl 6-methyl-2-oxo-4-phenyl-1,2,3,4-tetrahydropyrimidine-5-carboxylate (**8a**)

M.P. 228 °C; IR: 3179, 3157 (2 NH), 1739 (C=O ester), 1651 (C=O cyclic) cm^−1^; ^1^H NMR (DMSO-*d_6_*): δ 10.46, 9.84 (s, 2H, 2NH exchanged by D_2_O), 7.78–7.14 (m, 4H, CH_arom_), 5.42 (s, 1H, CH_Cyclic_), 4.7 (s, 3H, CH_3_), 2.35 (s, 3H, CH_3Pyrimidinium_); ^13^C NMR (DMSO-*d_6_*): δ 176.52, 163.78, 145.44, 143.91, 129.08, 128.11, 126.87, 101.30, 60.46, 54.58. (*Anal*. Calcd. for C_13_H_14_N_2_O_3_ (246.26): C, 63.40; H, 5.73; N, 11.38. Found: C, 63.28; H, 5.82; N, 11.24.

#### 2.1.14. Methyl 4-(4-chlorophenyl)-6-methyl-2-oxo-1,2,3,4-tetrahydropyrimidine-5-carboxylate (**9a**)

M.P. 224 °C; IR: 3133, 3115 (2 NH), 1725 (C=O ester), 1641 (C=O cyclic) cm^−1^; ^1^H NMR (DMSO-*d_6_*): δ 10.15, 9.04 (s, 2H, 2NH exchanged by D_2_O), 7.39–7.06 (m, 4H, CH_arom_), 5.08 (s, 1H, CH_Cyclic_), 3.92 (s, 3H, OCH_3_), 2.17 (s, 3H, CH_3Pyrimidinium_); ^13^C NMR (DMSO-*d_6_*): δ 171.13, 161.27, 144.41, 142.47, 129.81, 128.06, 127.19, 105.23, 64.17, 54.15. (*Anal*. Calcd. for C_13_H_13_ClN_2_O_3_ (280.70): C, 55.62; H, 4.67; N, 9.89 Found: C, 55.55; H, 4.51; N, 10.05.

#### 2.1.15. Methyl 6-methyl-4-(4-nitrophenyl)-2-oxo-1,2,3,4-tetrahydropyrimidine-5-carboxylate (**10a**)

M.P. 236 °C; IR: 3184, 3112 (2 NH), 1751 (C=O ester), 1657 (C=O cyclic) cm^−1^; ^1^H NMR (DMSO-*d_6_*): δ 10.15, 9.58 (s, 2H, 2NH exchanged by D_2_O), 7.99–7.18 (m, 4H, CH_arom_), 5.1 (s, 1H, CH_Cyclic_), 4.01 (s, 3H, OCH_3_), 2.25 (s, 3H, CH_3Pyrimidinium_); ^13^C NMR (DMSO-*d_6_*): δ 175.63, 167.12, 144.44, 143.44, 129.57, 128.74, 126.27, 101.74, 60.89, 54.48. (*Anal*. Calcd. for C_13_H_13_N_3_O_5_ (291.25): C, 53.61; H, 4.50; N, 14.43. Found: C, 53.73; H, 4.46; N, 14.21.

#### 2.1.16. Methyl 4-(4-methoxyphenyl)-6-methyl-2-oxo-1,2,3,4-tetrahydropyrimidine-5-carboxylate (**11a**)

M.P. 173 °C; IR: 3181, 3127 (2 NH), 1754 (C=O ester), 1661 (C=O cyclic) cm^−1^; ^1^H NMR (DMSO-*d_6_*): δ 10.72, 8.99 (s, 2H, 2NH exchanged by D_2_O), 7.75–7.14 (m, 4H, CH_arom_), 5.14 (s, 1H, CH_Cyclic_), 4.13 (s, 3H, COOCH_3_), 3.88 (s, 3H, PhOCH_3_), 2.30 (s, 3H, CH_3Pyrimidinium_); ^13^C NMR (DMSO-*d_6_*): δ 177.81, 164.67, 147.25, 141.95, 129.78, 128.74, 126.23, 111.21, 69.47, 54.57. (*Anal*. Calcd. for C_14_H_16_N_2_O_4_ (276.28): C, 60.86; H, 5.84; N, 10.14. Found: C, 60.76; H, 5.61; N, 10.41.

#### 2.1.17. Methyl 4-[4-(dimethylamino)phenyl]-6-methyl-2-oxo-1,2,3,4-tetrahydropyrimidine-5-carboxylate (**12a**)

M.P. 215 °C; IR: 3189, 3115 (2 NH), 1721 (C=O ester), 1648 (C=O cyclic) cm^−1^; ^1^H NMR (DMSO-*d_6_*): δ 10.78, 9.16 (s, 2H, 2NH exchanged by D_2_O), 8.01–7.21 (m, 4H, CH_arom_), 5.29 (s, 1H, CH_Cyclic_), 4.25 (s, 3H, PhOCH_3_), 4.11 (s, 3H, COOCH_3_), 2.31 (s, 3H, CH_3Pyrimidinium_); ^13^C NMR (DMSO-*d_6_*): δ 175.21, 164.67, 145.99, 143.21, 129.78, 128.97, 126.81, 101.74, 60.85, 54.14. (*Anal*. Calcd. for C_15_H_19_N_3_O_3_ (289.33): C, 62.27; H, 6.62; N, 14.52. Found: C, 66.13; H, 6.49; N, 14.39.

#### 2.1.18. Methyl 4-(4-hydroxyphenyl)-6-methyl-2-oxo-1,2,3,4-tetrahydropyrimidine-5-carboxylate (**13a**)

M.P. 256 °C; IR: 3198, 3141 (2 NH), 1728 (C=O ester), 1664 (C=O cyclic) cm^−1^; ^1^H NMR (DMSO-*d_6_*): δ 10.78, 9.65 (s, 2H, 2NH exchanged by D_2_O), 8.05 (s, 1H, OH exchanged by D_2_O), 7.84–7.02 (m, 4H, CH_arom_), 5.41 (s, 1H, CH_Cyclic_), 4.12 (s, 3H, OCH_3_), 2.34 (s, 3H, CH_3Pyrimidinium_); ^13^C NMR (DMSO-*d_6_*): δ 176.81, 168.14, 145.89, 143.74, 129.95, 128.42, 126.75, 101.37, 60.48, 54.84. (*Anal*. Calcd. for C_13_H_14_N_2_O_4_ (262.26): C, 59.54; H, 5.38; N, 10.68. Found: C, 59.22; H, 5.41; N, 10.72.

#### 2.1.19. Methyl 6-methyl-4-phenyl-2-thioxo-1,2,3,4-tetrahydropyrimidine-5-carboxylate (**14b**)

M.P. 228 °C; IR: 3175, 3135 (2 NH), 1731 (C=O ester) cm^−1^; ^1^H NMR (DMSO-*d_6_*): δ 11.03, 9.74 (s, 2H, 2NH exchanged by D_2_O), 7.71–7.35 (m, 5H, CH_arom_), 5.20 (s, 1H, CH_Cyclic_), 4.00 (s, 3H, OCH_3_), 2.30 (s, 3H, CH_3Pyrimidinium_); ^13^C NMR (DMSO-*d_6_*): δ 177.52, 167.14, 146.32, 143.45, 129.79, 128.38, 126.79, 101.64, 61.97, 54.75. (*Anal*. Calcd. for C_13_H_14_N_2_O_2_S (262.32): C, 59.52; H, 5.38; N, 10.68. Found: C, 59.74; H, 5.24; N, 10.43.

#### 2.1.20. Methyl 4-(4-chlorophenyl)-6-methyl-2-thioxo-1,2,3,4-tetrahydropyrimidine-5-carboxylate (**15b**)

M.P. 209 °C; IR: 3160, 3141 (2 NH), 1725 (C=O ester) cm^−1^; ^1^H NMR (DMSO-*d_6_*): δ 11.08, 9.35 (s, 2H, 2NH exchanged by D_2_O), 7.84–7.61 (m, 4H, CH_arom_), 5.24 (s, 1H, CH_Cyclic_), 3.87 (s, 3H, OCH_3_), 2.38 (s, 3H, CH_3Pyrimidinium_); ^13^C NMR (DMSO-*d_6_*): δ 176.14, 167.32, 145.67, 144.49, 125.47, 128.47, 126.94, 101.31, 60.45, 54.61. (*Anal*. Calcd. for C_13_H_13_ClN_2_O_2_S (296.77): C, 52.61; H, 4.42; N, 9.44. Found: C, 52.48; H, 4.21; N, 9.19.

#### 2.1.21. Methyl 6-methyl-4-(4-nitrophenyl)-2-thioxo-1,2,3,4-tetrahydropyrimidine-5-carboxylate (**16b**)

M.P. 207 °C; IR: 3192, 3161 (2 NH), 1725 (C=O ester) cm^−1^; ^1^H NMR (DMSO-*d_6_*): δ 10.95, 9.14 (s, 2H, 2NH exchanged by D_2_O), 7.65-7.15 (m, 4H, CH_arom_), 5.03 (s, 1H, CH_Cyclic_), 4.15 (s, 3H, OCH_3_), 2.36 (s, 3H, CH_3Pyrimidinium_); ^13^C NMR (DMSO-*d_6_*): δ 176.14, 167.32, 146.47, 146.84, 130.42, 129.17, 126.71, 110.29, 67.20, 54.78. (*Anal*. Calcd. for C_13_H_13_N_3_O_4_S (307.32): C, 50.81; H, 4.24; N, 13.67. Found: C, 50.65; H, 4.31; N, 13.57.

#### 2.1.22. Methyl 4-(4-methoxyphenyl)-6-methyl-2-thioxo-1,2,3,4-tetrahydropyrimidine-5-carboxylate (**17b**)

M.P. 201 °C; IR: 3145, 3114 (2 NH), 1728 (C=O ester) cm^−1^; ^1^H NMR (DMSO-*d_6_*): δ 10.43, 9.74 (s, 2H, 2NH exchanged by D_2_O), 7.67–7.01 (m, 4H, CH_arom_), 5.24 (s, 1H, CH_Cyclic_), 4.12 (s, 3H, COOCH_3_), 3.89 (s, 3H, PhOCH_3_), 2.31 (s, 3H, CH_3Pyrimidinium_); ^13^C NMR (DMSO-*d_6_*): δ 174.14, 165.24, 145.89, 143.61, 129.75, 128.64, 126.37, 101.89, 60.64, 54.85. (*Anal*. Calcd. for C_14_H_16_N_2_O_3_S (292.35): C, 57.52; H, 5.52; N, 9.58. Found: C, 57.61; H, 5.41; N, 9.42.

#### 2.1.23. Methyl 4-[4-(dimethylamino)phenyl]-6-methyl-2-thioxo-1,2,3,4-tetrahydropyrimidine-5-carboxylate (**18b**)

M.P. 179 °C; IR: 3159, 3147 (2 NH), 1737 (C=O ester) cm^−1^; ^1^H NMR (DMSO-*d_6_*): δ 10.95, 9.17 (s, 2H, 2NH exchanged by D_2_O), 7.85–7.18 (m, 4H, CH_arom_), 5.35 (s, 1H, CH_Cyclic_), 4.85 (s, 6H, N(CH_3_)_2_), 4.19 (s, 3H, OCH_3_), 2.57 (s, 3H, CH_3Pyrimidinium_); ^13^C NMR (DMSO-*d_6_*): δ 176.81, 165.84, 146.17, 143.27, 130.94, 128.84, 126.67, 101.15, 60.84, 54.69. (*Anal*. Calcd. for C_15_H_19_N_3_O_2_S (305.39): C, 58.99; H, 6.27; N, 13.76. Found: C, 59.17; H, 6.16; N, 13.49.

#### 2.1.24. Methyl 4-(4-hydroxyphenyl)-6-methyl-2-thioxo-1,2,3,4-tetrahydropyrimidine-5-carboxylate (**19b**)

M.P. 253 °C; IR: 3184, 3191 (2 NH), 1750 (C=O ester) cm^−1^; ^1^H NMR (DMSO-*d_6_*): δ described by, 9.89 (s, 2H, 2NH exchanged by D_2_O), 8.42 (s, 1H, OH exchanged by D_2_O), 7.30–7.04 (m, 4H, CH_arom_), 5.41 (s, 1H, CH_Cyclic_), 4.18 (s, 3H, OCH_3_), 2.24 (s, 3H, CH_3Pyrimidinium_); ^13^C NMR (DMSO-*d_6_*): δ 175.81, 165.67, 148.41, 142.78, 128.64, 128.15, 126.04, 101.74, 59.41, 54.71. (*Anal*. Calcd. for C_13_H_14_N_2_O_2_S (287.32): C, 56.10; H, 5.10; N, 10.06. Found: C, 56.00; H, 5.23; N, 9.94.

### 2.2. Pumice Sampling and Sample Preparation

Ten pumice samples were collected from the Abu Treifiya Basin, in the Eastern desert of Egypt. The samples were crushed and ground to reduce the size to 150 meshes for mineralogical and chemical analysis. In addition, six hand samples collected from the field were chosen and prepared for thin section studies. The chemical analysis of the volcanic rocks was performed using X-ray fluorescence spectrometry (XRF), and the crystal structure was achieved using X-ray diffraction (XRD). Surface area, pore volume, and pore size distribution were then calculated.

The study of the textural properties of pumice samples involves measuring textural parameters, such as surface area, pore volume, porosity, and pore size. The porosity of pumice samples was estimated using the saturation (or imbibition) method described by Lawrence et al. [31] using the following equation:

(1) $$Ø=\frac{Vbulk-Vmatrix}{Vbulk}=\frac{\left(Wsat-Wdry\right)/\rho \mathrm{fluid}}{Vbulk}$$

where (*W_sat_*) is the weight of the saturated sample, (*W_dry_*) is the weight of dry samples, (ρ_fluid_) is the density of the saturating fluid, and (*V_bulk_*) is the bulk volume of the sample.

**Figure 1:**
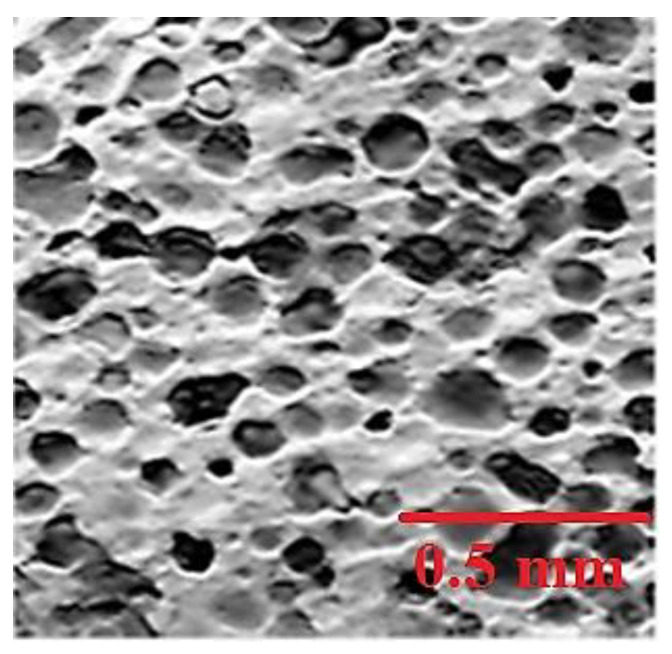
Photomicrograph of pumice sample showing pore size and distribution taken by binakuler microscope.

A surface and cross-section were prepared for the pumice’s pore size distribution. Micrographs were taken on a Nikon binocular microscope supported by a high-resolution digital canon camera; the micrographs were taken at a magnification ranging from 40× to 60× Figure 1. The images were manually corrected using Photoshop CS5 (Adobe) to remove any dark parts and obvious debris. Pore count and pore size were determined in 1 cm^2^ using a particular counting stage.

Moreover, the micrographs stated that most vesicles in pumice are interconnected. Accordingly, the pore surface area of the present pumice (A) is given by A = 4 V/w, where V is the pore volume, and w is the width (diameter) [32].

## 3. Results and Discussion

### 3.1. Chemistry

In recent years, there have been continuous demands for implementing organic reactions under eco-friendly conditions. On the other hand, synthetic manipulations are usually preferred when using non-hazardous chemicals and avoiding toxic organic solvents. Moreover, in industrial processes, there is an urgent need to replace toxic solvents with green, as a tremendous amount of solvent gets wasted.

The heterogeneous catalyst pumice in this context is interesting. It is cheap, eco-friendly with a non-toxic nature, easily handled and operated, and thermally stable. Furthermore, the reaction conditions’ mildness attracted luminaries’ attention for its applications in organic synthesis.

**Scheme 1:**
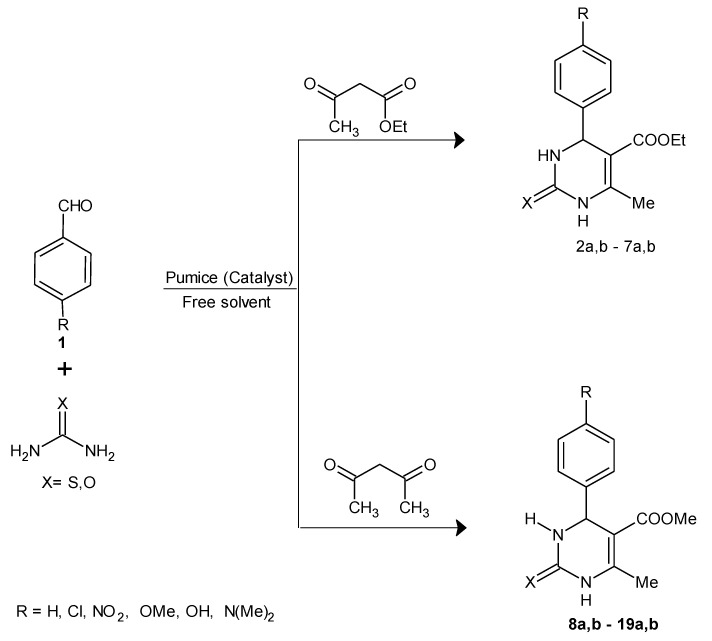
Synthesis of 3,4-dihydropyrimidine-2(1*H*)-ones/thiones.

We studied the catalyst amount effect on the synthesis of 3,4-dihydropyrimidin-2(1H)-ones/thiones (Scheme 1). Heating aromatic aldehydes (e.g., benzaldehyde, *p*-chlorobenzaldehyde, *p*-nitrobenzaldehyde, *p*-methoxybenzaldehyde, *N*,*N*-dimethylaminobenzaldehyde, and 4-hydroxybenzaldehyde) with urea/thiourea and ethyl acetoacetate or acetylacetone in the presence of a different amount of pumice (0.10–0.50 g) afforded the corresponding 3,4-dihydropyrimidine-2(1*H*)-ones/thiones an excellent yield within a short time (2–3 min). It is worth noting that the problems associated with toxic solvent usage (safety, pollution, and cost) were avoided in the conventional protocol. The optimized results are summarized in Table 1. The use of 0.40 g of pumice afforded 98% yield. On the other hand, increasing the amount of pumice catalyst to 0.50 g did not affect the yield (Table 1).

**Figure 2:**
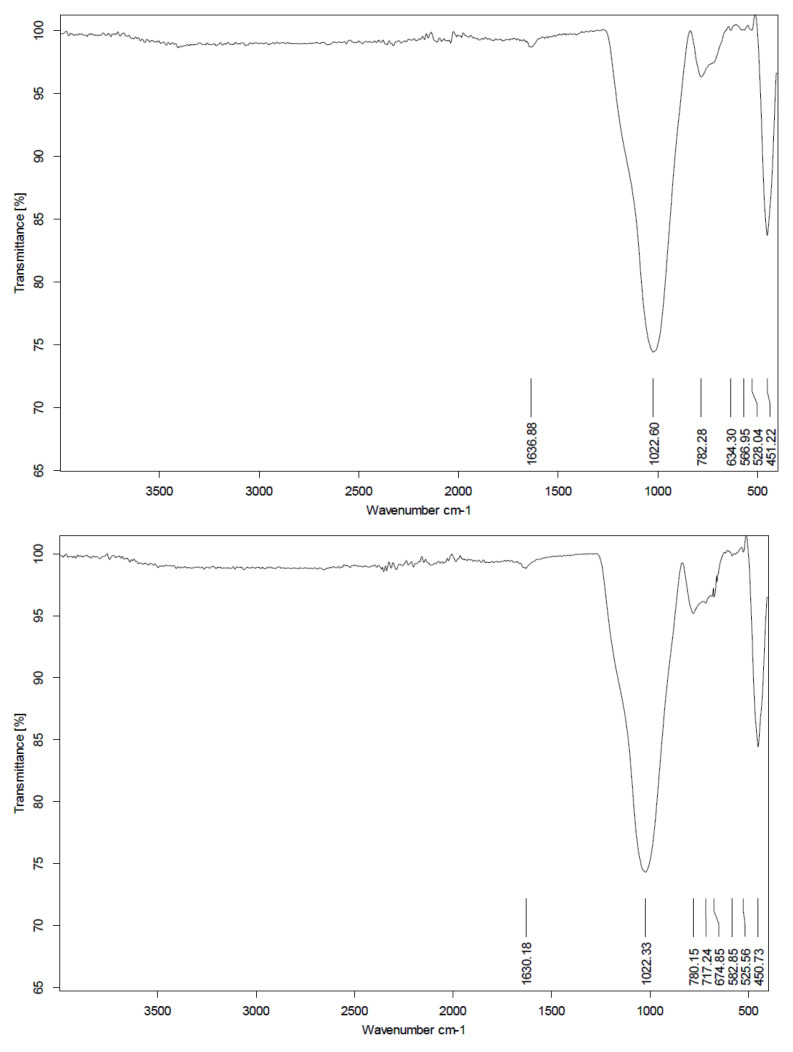
IR spectra of pumice (**A**) before reaction and (**B**) after reaction for five-time reaction cycles.

After the completion of the reaction, the catalyst was recovered quickly by heating the reaction mixture in ethanol and filtration. It was successfully reused without losing its catalytic activities or its amounts. The catalyst’s effectiveness was estimated, and it was found that it is effective in up to five reaction cycles. Indeed, its IR spectrum was not changed after five-time reaction cycles (Figure 2A,B, and Table 2).

**Scheme 2:**
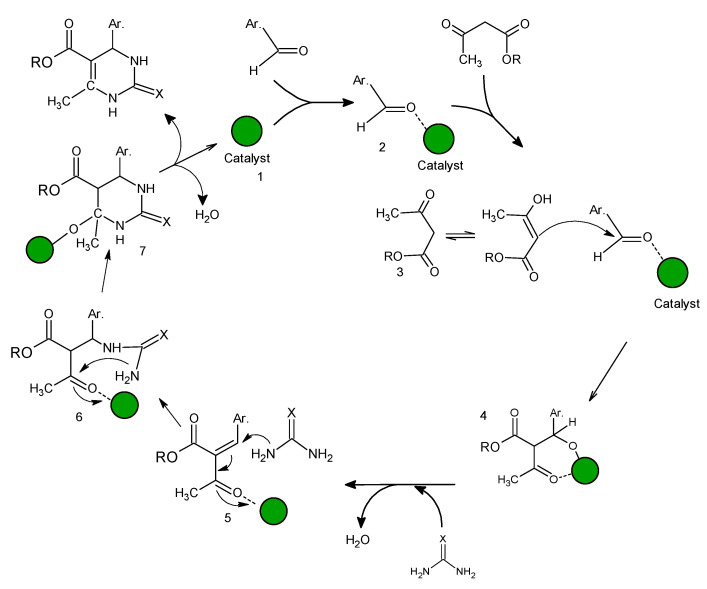
Reaction mechanism for the formation of 3,4-dihydropyrimidine-2-(1*H*)-ones/thiones.

The proposed reaction mechanism begins with the activation of aromatic aldehyde 2 by the catalyst **1**, which has a prominent acidic character (pumice is a volcanic rock consisting of 70% SiO_2_ and 13% Al_2_O_3_). Subsequent addition of ethyl 3-oxobutanoate is associated with H_2_O elimination and formation of adduct **4** facilitated by interchelation with catalyst. Urea or thiourea is then added to form C–N bond **5**; after that, inter nucleophilic attack of NH_2_ to C=O of CH_3_C=O **6**. The final step includes the catalyst separation with subsequent dehydration from the target compound. At this stage, the catalyst is free to restart the process again (Scheme 2).

Table 2 shows the effect of pumice amount on the reaction yield. Using 0.40 g of pumice afforded the best yield (up to 98%). On the other hand, increasing the amount of pumice catalyst to 0.50 g did not affect the yields (Table 2). This method is superior to the conventional procedure for the synthesis of 3,4-dihydropyrimidine-2-(1*H*)-one/thione derivatives by the simple green chemistry procedure. Furthermore, the dominant values of the conversion and selectivity percent confirmed the catalytic efficiency towards the preparation of the -one or -thione entries.

### 3.2. Characterization of Pumice Samples

**Figure 3:**
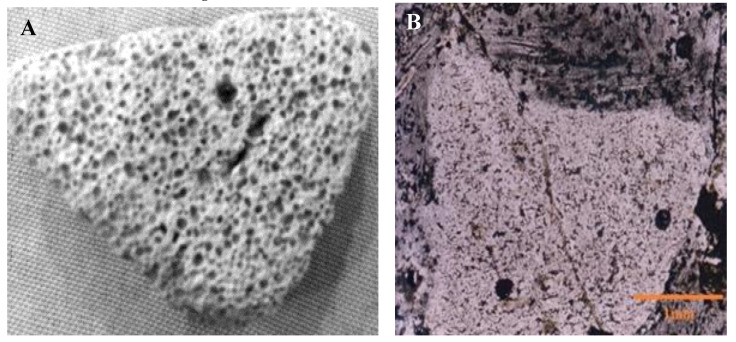
Photograph of pumice samples. (**A**) Photograph of a hand-specimen; (**B**) photomicrograph under a transmitted microscope.

Pyroclastic rocks represent pumice in the Abu Treifiya Basin; these volcaniclastics are a few tens of meters thick and overlie basaltic lava flows [42]. The pumice-bearing rocks comprise a well-bedded tuff. They are composed of angular, matrix- to clast-supported pumice in a vitric ash matrix (Figure 3). Pumice clasts range from about 7 cm to 30 cm in width, and most of the large volcanic clasts are broken into smaller clasts indicating in situ fragmentation.

#### 3.2.1. The Chemical Composition

**Figure 4:**
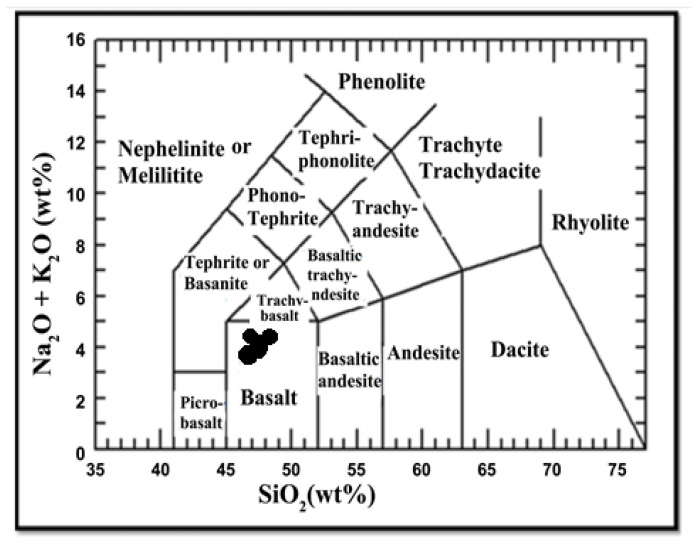
Plotting SiO_2_ vs. Na_2_O + K_2_O diagrams, showing the basaltic composition of pumice samples.

The geochemical composition [44,45] of four pumice samples is presented in Table 3. The chemical analysis indicated that SiO_2_ and Al_2_O_3_ were the main contents. The chemical analyses suggest they are basaltic in composition according to the total Na_2_O + K_2_O-SiO_2_ diagram Figure 4.

#### 3.2.2. X-ray Diffraction

**Figure 5:**
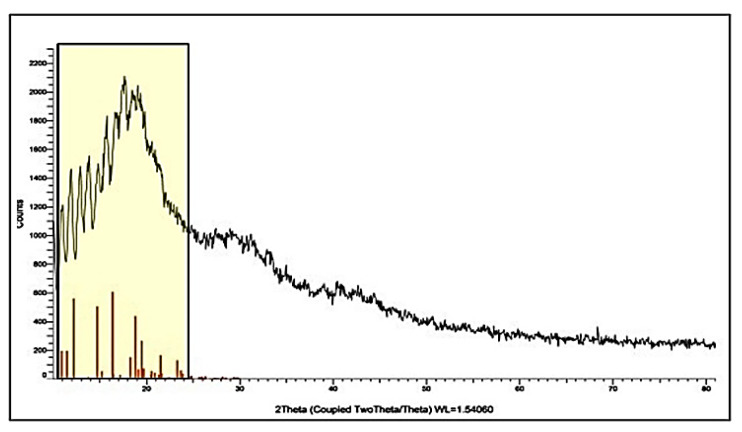
X-ray diffraction pattern of pumice rock.

XRD patterns of volcanic rocks show their crystal structure by observing the presence of both amorphous and crystalline phases [46]. The X-ray patterns of pumice samples in the present study (Figure 5) showed that they are amorphous materials. XRD analysis and appeared numbers of peaks are present at d-spacing 2.974 (80) 3.964 (55), which belong to Clinoptilolite mineral, and at d-spacing 5.096 (70) 3.420 (70), which belong to Heulandite mineral. These two minerals are the most common natural zeolites; they form well-developed crystals in veins, cavities, and vugs of volcanic rocks (pumice) or fine-grained crystals, mainly in volcaniclastics. The crystal structure of clinoptilolite and heulandite has a 3-dimensional aluminosilicate framework, which causes the development of micropores and channels [47]. More information about porosity and channel windows in the heulandite and clinoptilolite minerals is achieved by Baerlocher et al. [48].

#### 3.2.3. Physical Parameters

The textural parameters measuring pumice rock samples (surface area, pore volume, porosity, and pore size) are presented in Table 4. The pumice samples’ porosity ranges from 78.2–83.9% (by volume). The air bubbles created during its formation generates this high porosity. The samples are characterized by mesoporous to macroporous structure (pore size range from 21.1 to 64.5 nm) according to Thommes et al., 2015. In addition, the pumice samples also presented an average pore volume between 0.00531 and 0.00781 m^2^/g. The surface area of the pumice samples was between 0.053 and 1.47 m^2^/g. Thus, all indicators reveal that pumice has low density.

## 4. Conclusions

In conclusion, we have successfully developed a convenient, efficient, and rapid procedure for synthesizing 3,4-dihydropyrimidine-2-(1*H*)-one/thione derivatives via the one-pot multi-component condensation of aromatic aldehydes, urea/thiourea, and β-ketoesters employing pumice as a novel heterogeneous green catalyst. The chemical composition and characterization of the pumice catalyst were studied by XRD analysis. This protocol is eco-friendly, as it has proceeded under solvent-free conditions. Furthermore, this procedure tolerated a variety of 3,4-dihydropyrimidine-2-(1*H*)-one/thione derivatives under a simple, short time, non-tedious workup, and good yield procedure without any difficulties. Moreover, the catalyst can be reused up to five-time reaction cycles, and pure products were obtained in good to excellent quality. Notably, the present work revealed that pumice rock is a good heterogeneous porous catalyst. Its textural properties (surface area, pore volume, porosity, and pore size) play a crucial role in its catalytic activity.

## Figures and Tables

**Table 1:** Comparison amounts of pumice and yields, conversion, and selectivity % for Biginelli reactions under free solvent conditions.

Compound	Amount of Pumice						
	0.1 g	0.2g	0.3 g	0.4 g	0.5 g		
	Yield					Conversion % ^a^	Selectivity % ^b^
**2a**	58%	86%	93%	98%	98%	88	89.6
**3a**	53%	82%	95%	98%	98%	89.2	91
**4a**	61%	89%	92%	97%	97%	89.5	92.2
**5a**	57%	81%	97%	97%	97%	89	91.7
**6a**	64%	92%	98%	98%	98%	76.7	78.3
**7a**	48%	85%	91%	98%	98%	88.4	90.2
**2b**	70%	90%	96%	98%	98%	88.5	90.3
**3b**	56%	91%	95%	96%	96%	89.6	93.3
**4b**	63%	93%	94%	98%	98%	90	91.7
**5b**	72%	90%	97%	97%	97%	89.5	92.3
**6b**	61%	87%	96%	98%	98%	89.8	91.7
**7b**	58%	89%	95%	98%	98%	89.1	90.8
**8a**	72%	93%	97%	97%	97%	92.5	95.4
**9a**	60%	92%	97%	98%	98%	93.3	95.3
**10a**	59%	90%	96%	96%	96%	93.5	97.5
**11a**	55%	91%	97%	98%	98%	93.2	95.2
**12a**	71%	89%	97%	97%	97%	93.5	96.4
**13a**	73%	85%	92%	96%	96%	92.8	96.8
**14b**	51%	86%	97%	98%	98%	93	94.8
**15b**	58%	89%	94%	98%	98%	93.7	95.6
**16b**	59%	76%	89%	97%	97%	93.8	96.7
**17b**	67%	88%	96%	98%	98%	93.6	95.5
**18b**	68%	90%	94%	96%	96%	93.8	97.7
**19b**	60%	84%	92%	97%	97%	96.3	99.3

Reaction conditions: aromatic aldehyde (10 mmol), ethyl acetoacetate (15 mmol) or acetylacetone (15 mmol), urea (11 mmol) or thiourea (11 mmol), and pumice catalyst (0.1–0.5 g) was heated at 180°C for 1–3 min. ^a,b^ the conversion and selectivity percentages towards the different entries using the effective weight of pumice catalyst (0.4 g).

**Table 2:** Synthesis of 3,4-dihydropyrimidine-2(1*H*)-ones/thiones.

Entry		X	Y	M.P.	Reported
**2a**	OEt	O	H	202	201–202 [33]
**3a**	OEt	O	Cl	211	212–214 [34]
**4a**	OEt	O	NO_2_	209	209–210 [33]
**5a**	OEt	O	OMe	201	201–202 [33]
**6a**	OEt	O	N(Me)_2_	233	231–232 [33]
**7a**	OEt	O	OH	232	232–234 [34]
**2b**	OEt	S	H	208	207–209 [35]
**3b**	OEt	S	Cl	194	192–194 [36]
**4b**	OEt	S	NO_2_	207	207–208 [37]
**5b**	OEt	S	OMe	153	150–151 [34]
**6b**	OEt	S	N(Me)_2_	209	208–209 [33]
**7b**	OEt	S	OH	203	202–203 [33]
**8a**	OMe	O	H	228	232–234 [33]
**9a**	OMe	O	Cl	224	224–226 [35]
**10a**	OMe	O	NO_2_	236	236–238 [36]
**11a**	OMe	O	OMe	173	172 [37]
**12a**	OMe	O	N(Me)_2_	215	220 [38]
**13a**	OMe	O	OH	256	256 [39]
**14b**	OMe	S	H	228	228–230 [40]
**15b**	OMe	S	Cl	209	201 [41]
**16b**	OMe	S	NO2	213	212 [41]
**17b**	OMe	S	OMe	201	202 [41]
**18b**	OMe	S	N(Me)_2_	179	180 [42]
**19b**	OMe	S	OH	253	251–252 [43]

**Table 3:** Geochemical composition of pumice rock in (wt %).

SiO_2_	Al_2_O_3_	MgO	Na_2_O	CaO	Fe_2_O_3_	K_2_O	TiO_2_	MnO	L.O.I	Total
48.89	13.90	7.34	3.08	9.70	7.71	1.84	1.01	0.15	5.70	99.321
49.10	15.00	6.20	2.50	9.10	8.20	2.30	0.95	0.16	6.00	99.51
49.50	14.10	7.40	2.70	9.80	7.90	1.70	0.84	0.16	5.20	99.302
49.30	14.50	6.90	2.20	9.30	8.70	2.50	0.69	0.17	4.60	98.865

**Table 4:** The textural parameters measuring pumice rock samples (surface area, pore volume, porosity, and pore size).

No.	Bulk Porosity (%)	Average Pore Size nm	Average Pore Volume cm^2^ g^−1^	Average Surface Area
1	78.2	38.892	0.00661	0.6801
2	79.5	33.401	0.00631	0.7475
3	78.5	40.051	0.00531	0.05301
4	81.4	64.501	0.00551	0.4201
5	83.9	21.121	0.00781	1.47701

## Data Availability

The raw/processed data generated in this work are available upon request from the corresponding author.

## Supplementary Materials

The following supporting information can be downloaded at: https://www.mdpi.com/article/10.3390/molecules27186044/s1, Figure S1: 1H-NMR Spectrum of compound **2a**; Figure S2: 13C-NMR Spectrum of compound **2a**; Figure S3: Dept-135 Spectrum of compound **2a**; Figure S4: 1H-NMR Spectrum of compound **5a**; Figure S5: 13C-NMR Spectrum of compound **5a**; Figure S6: Dept-135 Spectrum of compound **5a**; Figure S7: 1H-NMR Spectrum of compound **2b**; Figure S8: 13C-NMR Spectrum of compound **2b**; Figure S9: Dept-135 Spectrum of compound **2b**.

## References

[1] Amer A.A., Abdelhamid A.A. (2017). One-Pot Multicomponent Synthesis of Some New Cyanopyridines. J. Heterocycl. Chem..

[2] Mohamed S.K., Simpson J., Marzouk A.A., Talybov A.H., Abdelhamid A.A., Abdullayev Y.A., Abbasov V.M. (2015). Multicomponent green synthesis, spectroscopic and structural investigation of multi-substituted imidazoles. Part 1. Z. Für Nat. B.

[3] Khalaf M.M., Abdelhamid A.A. (2016). Sol–gel derived mixed oxide zirconia: Titania green heterogeneous catalysts and their performance in acridine derivatives synthesis. Catal. Lett..

[4] Khalilov A.N., Abdelhamid A.A., Gurbanov A.V., Ng S.W. (2011). 9-(5-Bromo-2-hydroxyphenyl)-10-(2-hydroxypropyl)-3, 3, 6, 6-tetramethyl-1, 2, 3, 4, 5, 6, 7, 8, 9, 10-decahydroacridine-1, 8-dione. Acta Crystallogr. E.

[5] Khodairy A., Ali A.M., El-Wassimy M.T. (2017). Synthesis of Novel Chromene, Pyridine, Pyrazole, Pyrimidine, and Imidazole Derivatives via One-pot Multicomponent Reaction. J. Heterocycl. Chem..

[6] Bruckmann A., Krebs A., Bolm C. (2008). Organocatalytic reactions: Effects of ball milling, microwave and ultrasound irradiation. Green Chem..

[7] Rodríguez B., Bruckmann A., Rantanen T., Bolm C. (2007). Solvent-free carbon-carbon bond formations in ball mills. Adv. Synth. Catal..

[8] Raston C.L., Scott J.L. (2000). Chemoselective, solvent-free aldol condensation reaction. Green Chem..

[9] Shan Z.-X., Luo X.-X., Hu L., Hu X.-Y. (2010). New observation on a class of old reactions: Chemoselectivity for the solvent-free reaction of aromatic aldehydes with alkylketones catalyzed by a double-component inorganic base system. Sci. China Chem..

[10] Tanaka K., Toda F.K. (2000). Solvent-free organic synthesis. Chem. Rev..

[11] Schmeyers J., Toda F., Boy J., Kaupp G. (1998). Quantitative solid–solid synthesis of azomethines. J. Chem. Soc. Perkin Trans..

[12] Atwal K.S., Swanson B.N., Unger S.E., Floyd D.M., Moreland S., Hedberg A., Reilly B.C. (1991). Dihydropyrimidine calcium channel blockers. 3. 3-Carbamoyl-4-aryl-1, 2, 3, 4-tetrahydro-6-methyl-5-pyrimidinecarboxylic acid esters as orally effective antihypertensive agents. J. Med. Chem..

[13] Kappe C.O. (2000). Biologically active dihydropyrimidones of the Biginelli-type—a literature survey. Eur. J. Med. Chem..

[14] Brands M., Endermann R., Gahlmann R., Kruger J., Raddatz S. (2003). Dihydropyrimidinones—A new class of anti-staphylococcal antibiotics. Bioorg. Med. Chem. Lett..

[15] Peng J., Deng Y. (2001). Ionic liquids catalyzed Biginelli reaction under solvent-free conditions. Tetrahedron Lett..

[16] Arfan A., Paquin L., Bazureau J.P. (2007). Acidic task-specific ionic liquid as catalyst of microwave-assisted solvent-free Biginelli reaction. Russ. J. Org. Chem..

[17] Ahmed E.A., Mohamed M.A., El-Saghier A.M. (2012). One-pot synthesis of dihydropyrimidin-2 (1H)-ones catalyzed by ceric (IV) ammonium nitrate (CAN) under solvent free conditions. J. Am. Sci..

[18] Adibi H., Samimi H.A., Beygzadeh M. (2007). Iron (III) trifluoroacetate and trifluoromethanesulfonate: Recyclable Lewis acid catalysts for one-pot synthesis of 3, 4-dihydropyrimidinones or their sulfur analogues and 1, 4-dihydropyridines via solvent-free Biginelli and Hantzsch condensation protocols. Catal. Commun.

[19] Ma Y., Qian C., Wang L., Yang M. (2000). Lanthanide triflate catalyzed Biginelli reaction. One-pot synthesis of dihydropyrimidinones under solvent-free conditions. J. Org. Chem..

[20] Phukan M., Kalita M.K., Borah R. (2010). A new protocol for Biginelli (or like) reaction under solvent-free grinding method using Fe (NO3) 3.9 H2O as catalyst. Green Chem. Lett. Rev..

[21] Su W., Li J., Zheng Z., Shen Y. (2005). One-pot synthesis of dihydropyrimidiones catalyzed by strontium (II) triflate under solvent-free conditions. Tetrahedron Lett..

[22] Yilai Jiao X.F. (2020). Porous Materials for Catalysis: Toward Sustainable Synthesis and Applications of Zeolites. Sustainable Nanoscale Engineering from Materials Design to Chemical Processing.

[23] Qiao Z.A., Huo Q. (2011). Synthetic Chemistry of the Inorganic Ordered Porous Materials. Modern Inorganic Synthetic Chemistry.

[24] Liu P.S., Chen G.F. (2014). General introduction to porous materials Porous Materials. Porous Materials.

[25] Sepehr M.N., Zarrabi M., Kazemian H., Amrane A., Yaghmaian K., Ghaffari H.R. (2013). Removal of hardness agents, calcium and magnesium, by natural and alkaline modified pumice stones in single and binary systems. Appl. Surf. Sci..

[26] Tapan M., Yalçın Z., İçelli O., Kara H., Orak S., Özvan A., Depci T. (2014). Effect of physical, chemical and electro-kinetic properties of pumice samples on radiation shielding properties of pumice material. Ann. Nucl. Energy.

[27] Al-Naaymi T.A., Ali M.A. (2013). Chemical physical and geotechnical properties comparison between scoria and pumice deposits in Dhamar—Rada volcanic field—SW Yemen. Aust. J. Basic Appl. Sci..

[28] Ottanà R., Saija L.M., Burriesci N., Giordano N. (1982). Hydrothermal synthesis of zeolites from pumice in alkaline and saline environment. Zeolites.

[29] Dharma Rao G.B., Acharya B.N., Verma S.K., Kaushik M.P. (2011). N,N′-Dichlorobis (2, 4, 6-trichlorophenyl) urea (CC-2) as a new reagent for the synthesis of pyrimidone and pyrimidine derivatives via Biginelli reaction. Tetrahedron Lett..

[30] Dharma Rao G.B., Acharya B.N., Kaushik M.P. (2013). An efficient synthesis of β-ketoesters via transesterification and its application in Biginelli reaction under solvent-free, catalyst-free conditions. Tetrahedron Lett..

[31] Lawrence M.A., David R.C. (2015). Characterization and Analysis of Porosity and Pore Structures. Rev. Mineral. Geochem..

[32] Whitham A.G., Sparks R.S.J. (1986). Pumice. Bull. Volcanol..

[33] Tamaddon F., Razmi Z., Jafari A.A. (2010). Synthesis of 3, 4-dihydropyrimidin-2 (1H)-ones and 1, 4-dihydropyridines using ammonium carbonate in water. Tetrahedron Lett..

[34] Yadav J.S., Kumar S.P., Kondaji G., Rao R.S., Nagaiah K. (2004). A novel l-proline catalyzed Biginelli reaction: One-pot synthesis of 3, 4-dihydropyrimidin-2 (1 H)-ones under solvent-free conditions. Chem. Lett..

[35] Khaskel A., Gogoi P., Barman P., Bandyopadhyay B. (2014). Grindstone chemistry: A highly efficient and green method for synthesis of 3, 4-dihydropyrimidin-2-(1 H)-ones by l-tyrosine as an organocatalyst: A combined experimental and DFT study. RSC Adv..

[36] Safari J., Gandomi-Ravandi S. (2014). Carbon nanotubes supported by titanium dioxide nanoparticles as recyclable and green catalyst for mild synthesis of dihydropyrimidinones/thiones. J. Mol. Struct..

[37] Jetti S.R., Bhatewara A., Kadre T., Jain S. (2014). Silica-bonded N-propyl sulfamic acid as an efficient recyclable catalyst for the synthesis of 3, 4-dihydropyrimidin-2-(1H)-ones/thiones under heterogeneous conditions. Chin. Chem. Lett..

[38] Shirini F., Abedini M., Pourhasan-Kisomi R. (2014). N-Sulfonic acid poly (4-vinylpyridinium) chloride as a highly efficient and reusable catalyst for the Biginelli reaction. Chin. Chem. Lett..

[39] Sandeep P., Prashant B., Murlidhar P., Wadekarb S. (2017). Simple and efficient synthesis of 3,4-dihydropyrimidin-2(1H)-thiones utilizing l-proline nitrate as a proficient, recyclable and eco-friendly catalyst. J. Saudi Chem. Soc..

[40] Cepanec I., Litvić M., Filipan-Litvić M., Grüngold I. (2007). Antimony (III) chloride-catalysed Biginelli reaction: A versatile method for the synthesis of dihydropyrimidinones through a different reaction mechanism. Tetrahedron.

[41] Sibous S., Said B., Rachid G., Nouzha H., Amina H. (2017). Easy synthesis of 3, 4-dihydropyrimidin-2-(1H)-ones using phosphate fertilizers MAP, DAP AND TSP as efficient catalysts. J. Turkish Chem. Soc..

[42] Abhishek N., Vaibhav K., Dipak K. (2012). A facile approach for the synthesis of 3, 4-dihydropyrimidin-2-(1H)-ones using a microwave promoted Biginelli protocol in ionic liquid. J. Chem. Sci..

[43] Suresh P., Swati D., Sanjeevani Y. (2011). Pineapple juice as a natural catalyst for Biginelli reaction. Int. J. Org. Chem..

[44] Khalaf E.A., Abdel Motelib A., Hammed M.S., El Manawi A.H. (2015). Volcano-sedimentary characteristics in the Abu Treifiya Basin, Cairo-Suez District, Egypt: Example of dynamics and fluidization over sedimentary and volcaniclastic beds by emplacement of syn-volcanic basaltic rocks. J. Volcanol. Geotherm..

[45] Lebas M.J., Lemaitre R.W., Streckeisen A., Zanettin B. (1986). A chemical classification of volcanic rocks based on the total alkali-silica diagram. J. Petrol..

[46] Marantos I., Christidis G.E., Ulmanu M. (2011). Zeolite formation and deposits. Handb. Nat. Zeolites.

[47] Mansouri N., Rikhtegar N., Ahmad Panahi H., Atabi F. (2013). Porosity, Characterization and Structural Properties of Natural Zeolite-Clinoptilolite as a Sorbent. Environ. Prot. Eng..

[48] Baerlocher C., McCusker L.B., Olson D.H. (2007). Compendium of zeolite framework types: Building schemes and type characteristics. Atlas of Zeolite Structure Types.

