# Supplementary material for: Pumice as a Novel Natural Heterogeneous Catalyst for the Designation of 3,4-Dihydropyrimidine-2-(1H)-ones/thiones under Solvent-Free Conditions
Source: Molecules. 2022 Sep 16;27(18):6044. doi: 10.3390/molecules27186044 (PMC9503633; doi:10.3390/molecules27186044)
Supplement: Supplementary file 1 [file molecules-27-06044-s001.zip › molecules-1829113-supplementary.pdf]

molecules

MDPI

Supplementary Materials

Pumice as a Novel Natural Heterogeneous Catalyst for the Designation of 3,4-Dihydropyrimidine-2-(1H)-ones/thiones under Solvent-Free Conditions

Hany M. Abd El-Lateef [1,2,*], Mohamed Gouda [1,*], Mai M. Khalaf [1,2], Saad Shaaban [1,3], Nadia A. A. Elkanzi [4,5], El Sayed A. Saber [6], Antar A. Abdelhamid [2,7] and Ali M. Ali [2]

[1] Department of Chemistry, College of Science, King Faisal University, Al-Ahsa 31982, Saudi Arabia
[2] Department of Chemistry, Faculty of Science, Sohag University, Sohag 82534, Egypt
[3] Chemistry Department, Faculty of Science, Mansoura University, Mansoura 35516, Egypt
[4] Chemistry department, College of Science, Jouf University, Sakaka P.O. Box 2014, Saudi Arabia
[5] Chemistry Department, Faculty of Science, Aswan University, Aswan P.O. Box 81528, Egypt
[6] Geology Department, Faculty of Science, Sohag University, Sohag 82534, Egypt
[7] Chemistry Department, Faculty of Science, Albaha University, Albaha P.O. Box 1988, Saudi Arabia
* Correspondence: hmahmed@kfu.edu.sa or hany_shubra@science.sohag.edu.eg (H.M.A.E.-L.); mgoudaam@kfu.edu.sa (M.G.)

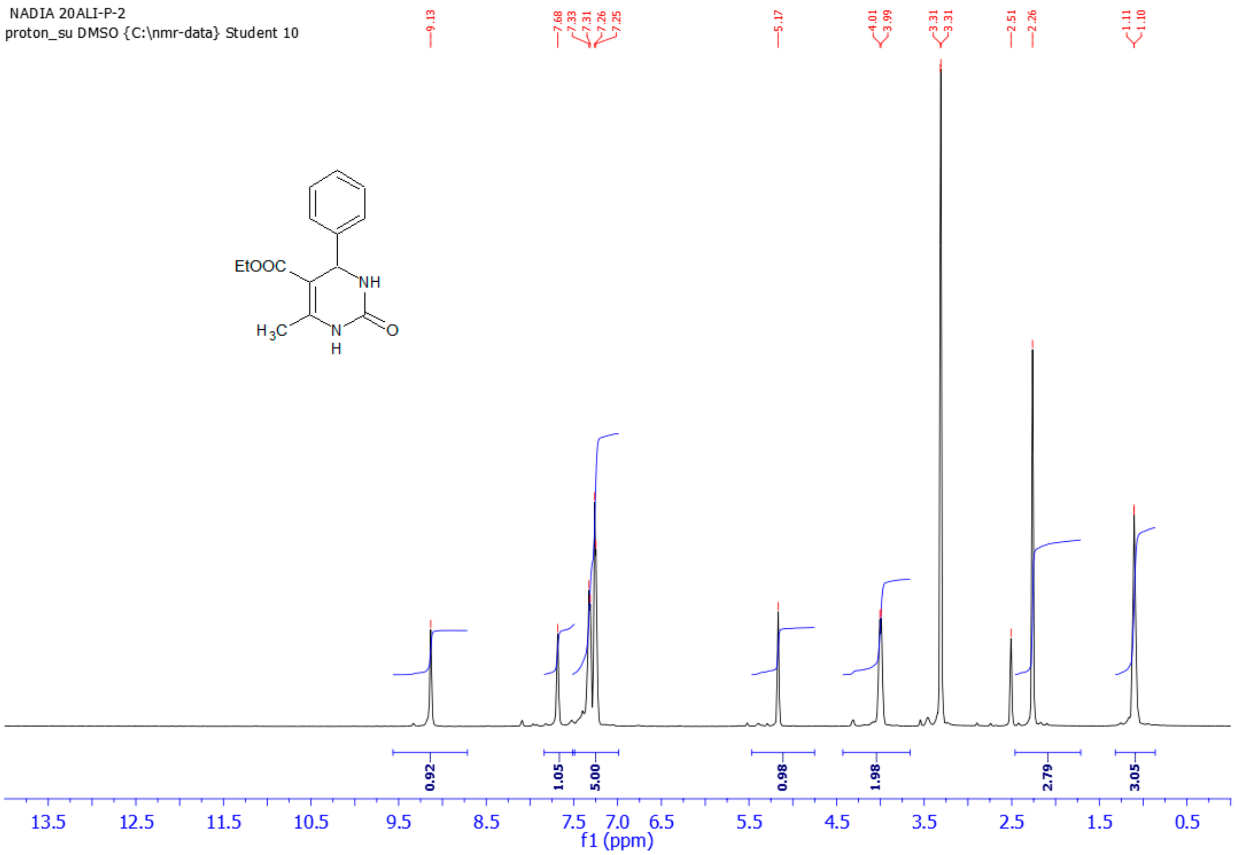

Figure S1. ^{1}H-NMR Spectrum of compound 2a.

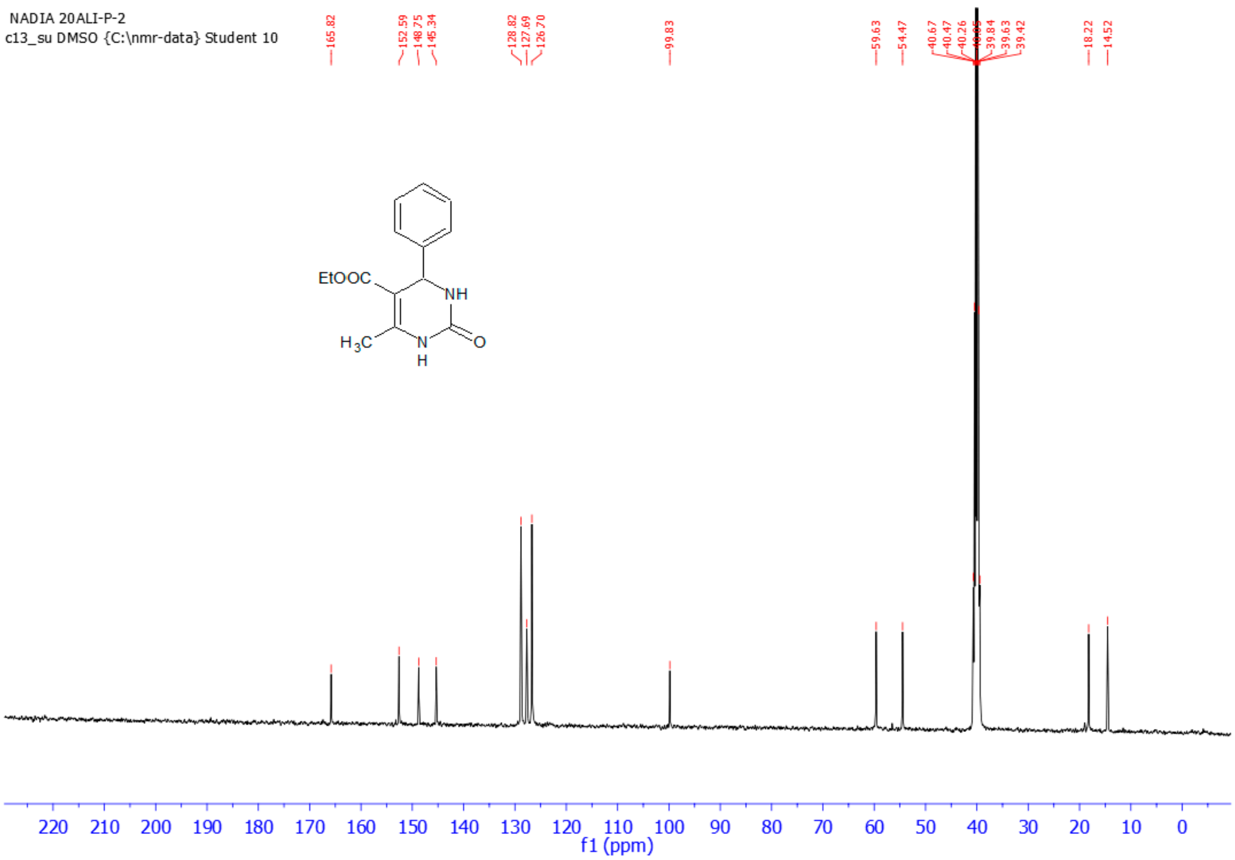
NADIA 20ALI-P-2
c13_su DMSO {C:\nmr-data} Student 10
165.82
152.59
148.75
145.34
128.82
127.69
126.70
99.83
59.63
54.47
18.22
14.52
EtOOC
NH
H3C
N
H
O
f1 (ppm)

Figure S2. ^{13}C-NMR Spectrum of compound 2a.

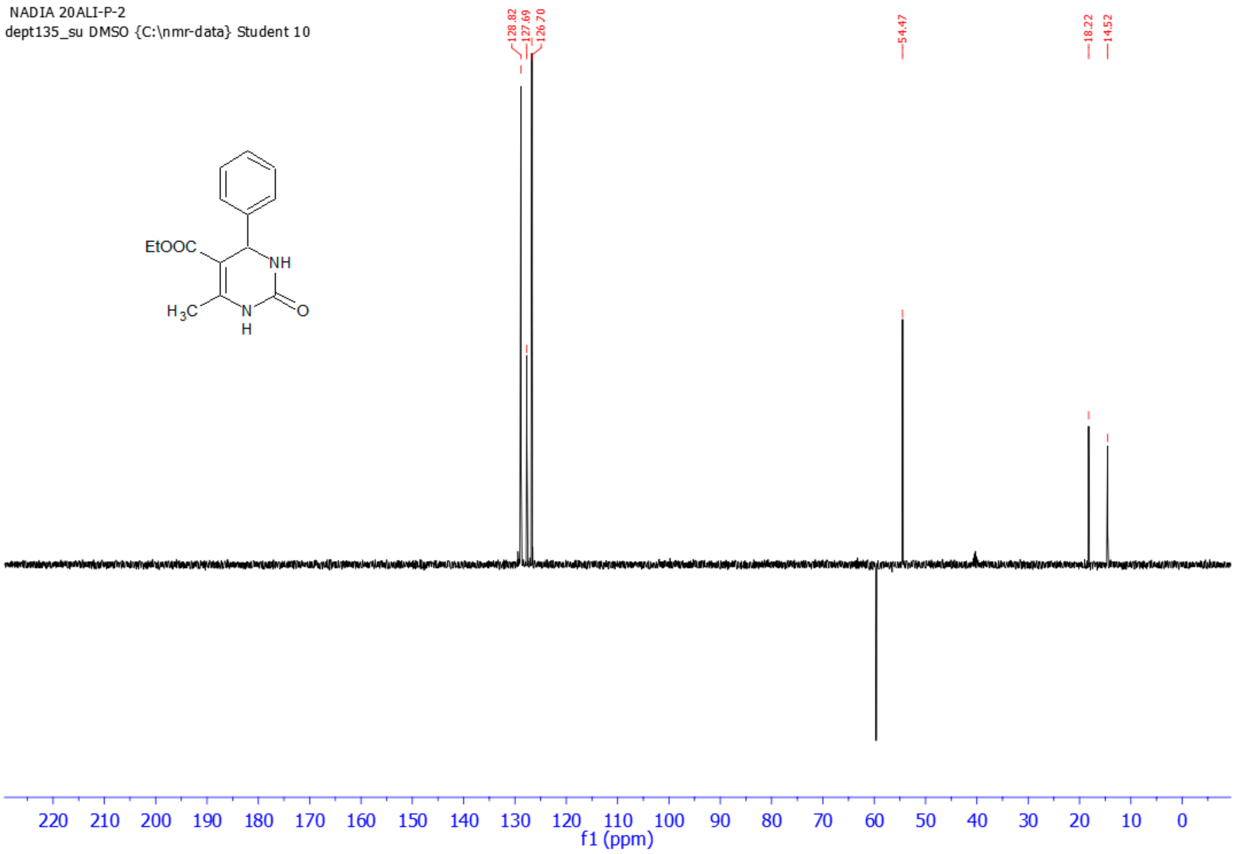

Figure S3. Dept-135 Spectrum of compound 2a.

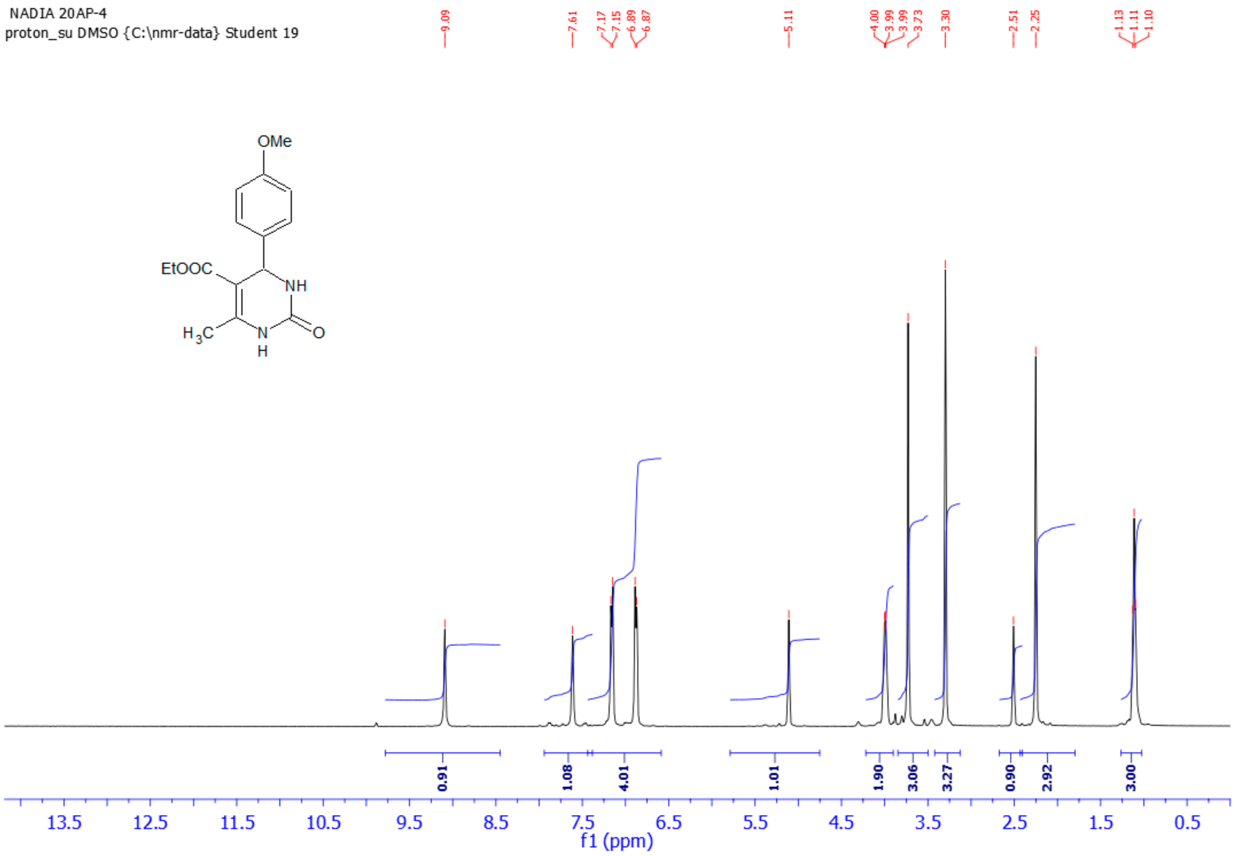

Figure S4. ^{1}H-NMR Spectrum of compound 5a.

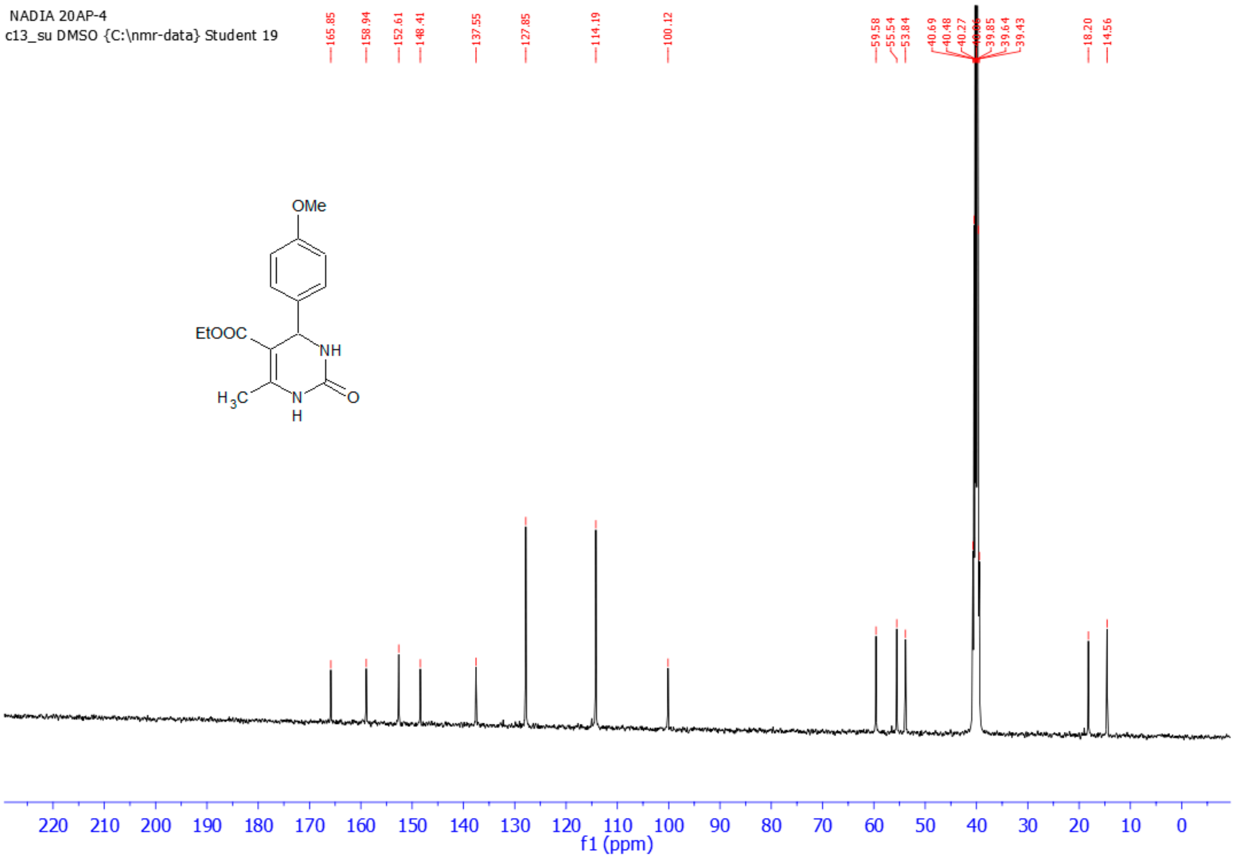

Figure S5. ^{13}C-NMR Spectrum of compound 5a.

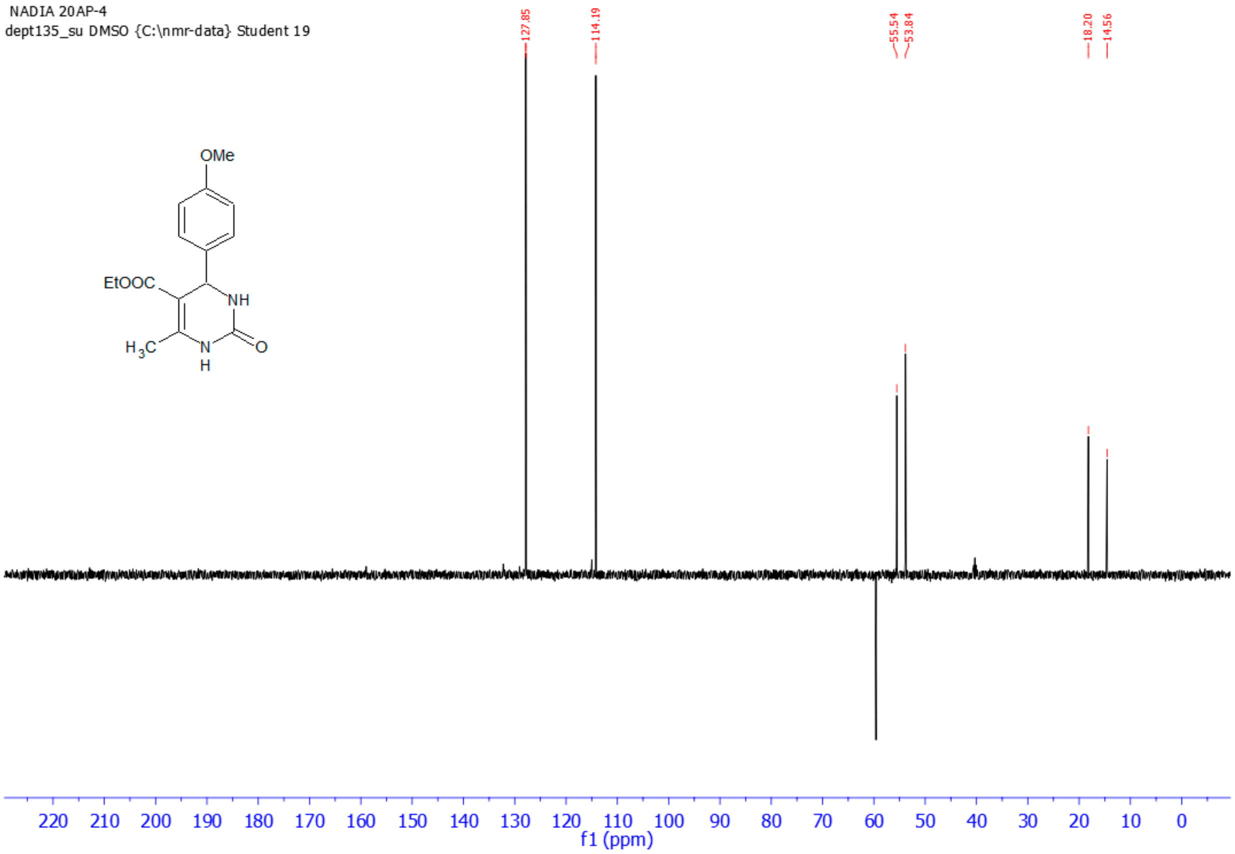

Figure S6. Dept-135 Spectrum of compound 5a.

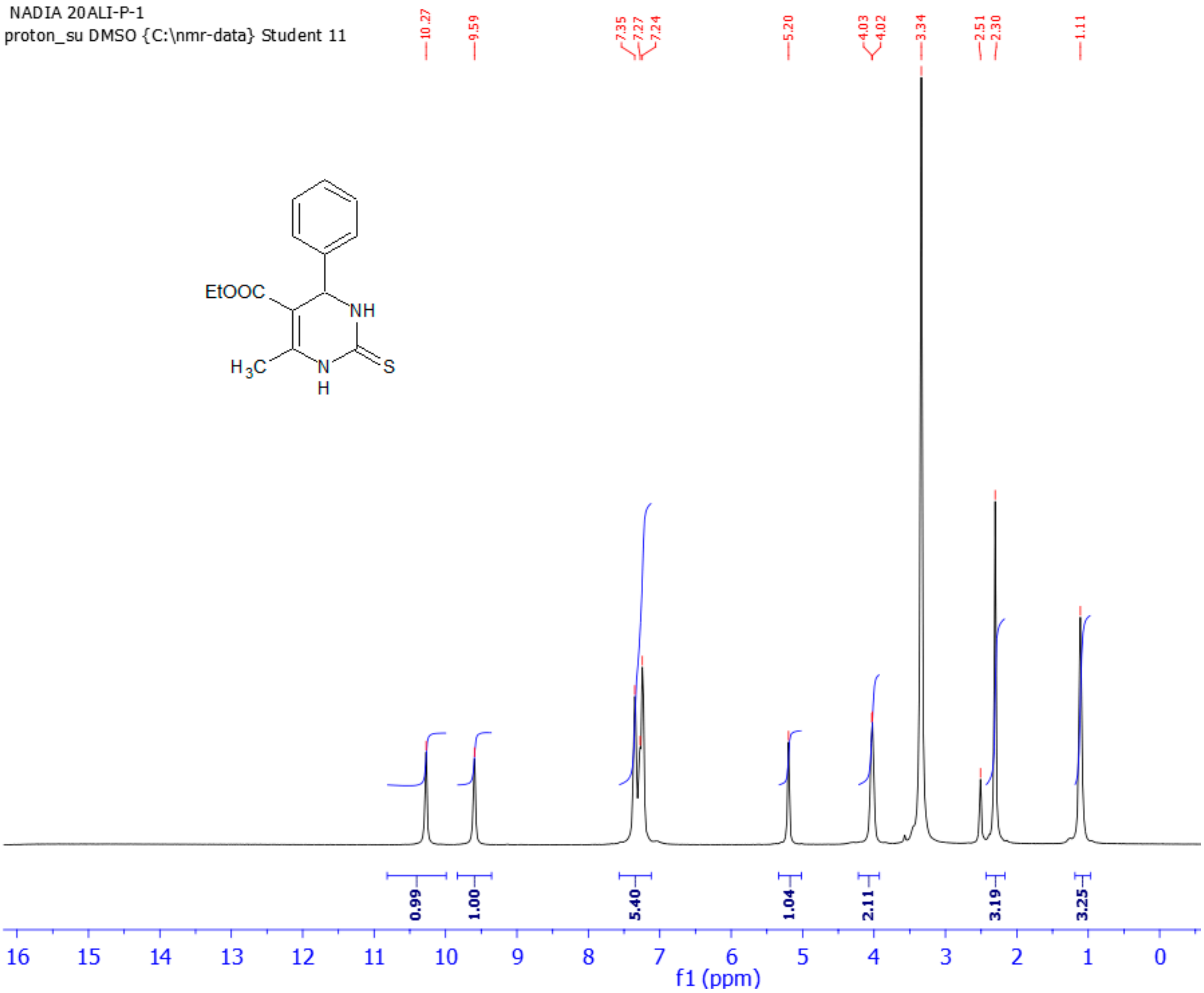

Figure S7. ^{1}H-NMR Spectrum of compound 2b.

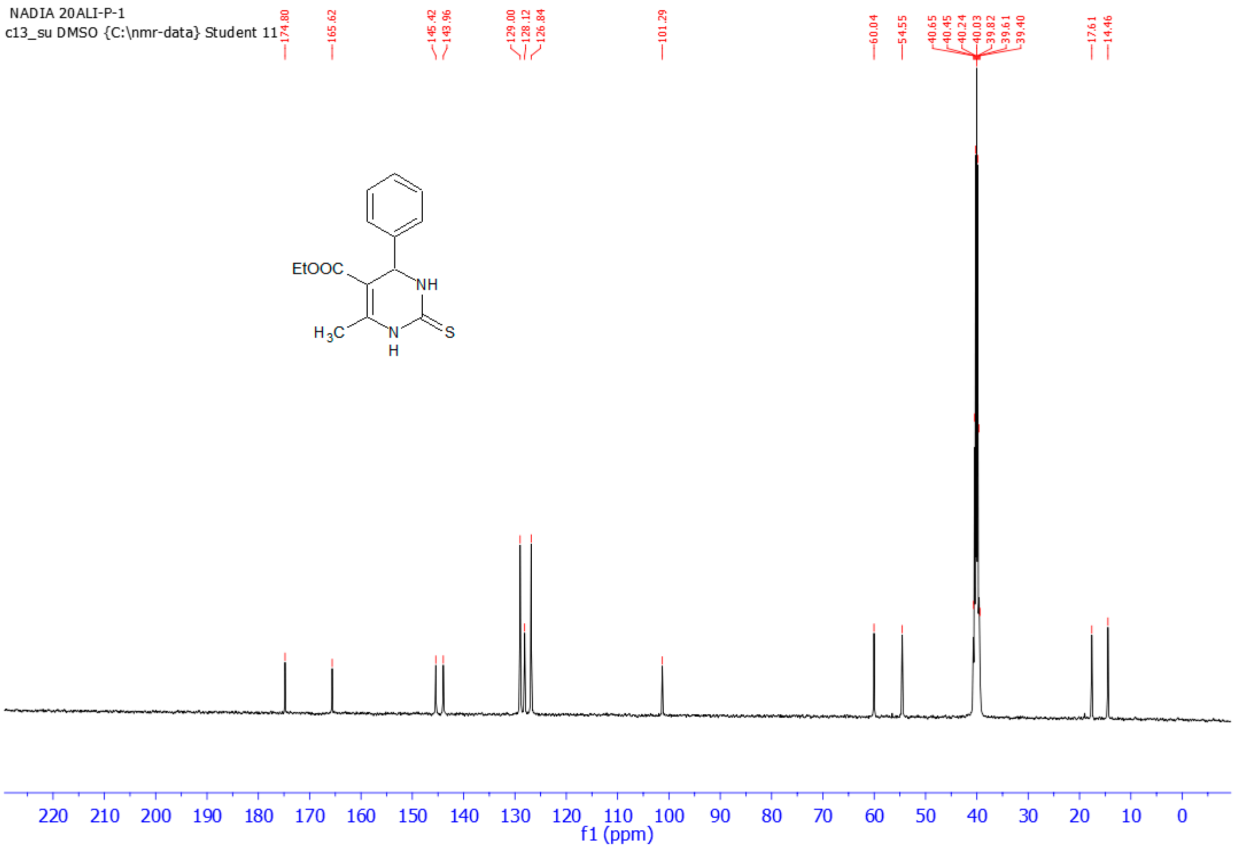

Figure S8. ^{13}C-NMR Spectrum of compound 2b.

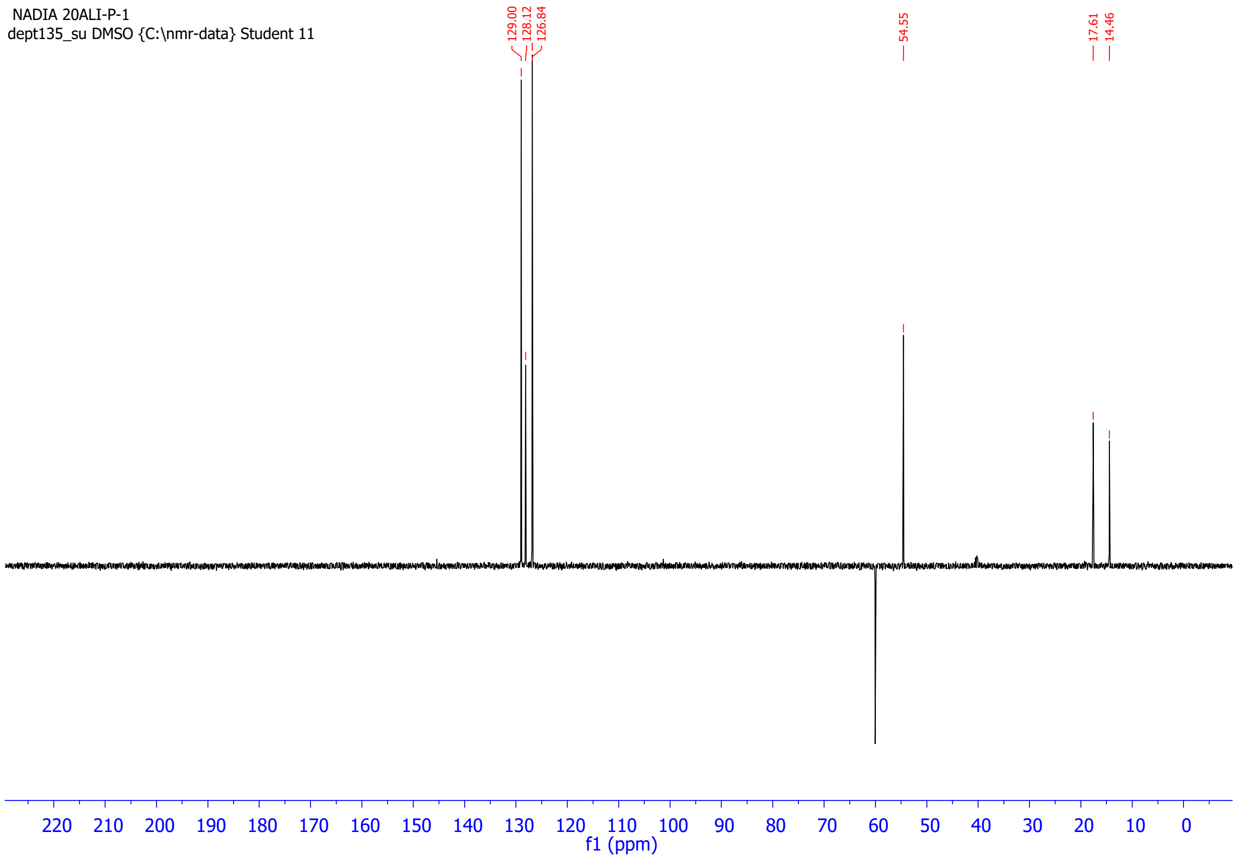

Figure S9. Dept-135 Spectrum of compound 2b.
